# Preliminary Experience with Virtual Monoenergetic Imaging and Iodine Mapping in the Primary Staging of Endometrial Cancer

**DOI:** 10.3390/cancers16061229

**Published:** 2024-03-20

**Authors:** Stephanie Tina Sauer, Henner Huflage, Sara Aniki Christner, Theresa Sophie Patzer, Matthias Kiesel, Anne Quenzer, Andreas Steven Kunz, Thorsten Alexander Bley, Jan-Peter Grunz

**Affiliations:** 1 Department of Diagnostic and Interventional Radiology, University Hospital Würzburg, Oberdürrbacher Straße 6, 97080 Würzburg, Germanyhuflage_h@ukw.de (H.H.); christner_s@ukw.de (S.A.C.); patzer_t@ukw.de (T.S.P.); kunz_a@ukw.de (A.S.K.); bley_t@ukw.de (T.A.B.); 2 Department of Obstetrics and Gynecology, University Hospital Würzburg, Josef-Schneider-Str. 4, 97080 Würzburg, Germany; kiesel_m2@ukw.de (M.K.);

**Keywords:** dual-energy computed tomography, endometrial cancer, virtual monoenergetic imaging, tumor conspicuity, iodine maps

## Abstract

**Simple Summary:**

This study investigates the efficacy of virtual monoenergetic images (VMIs) and iodine mapping from dual-energy CT in the primary staging of endometrial malignancies. In 21 women with endometrioid adenocarcinoma, VMIs at 40 keV demonstrated superior tumor contrast and contrast-to-noise ratios, enhancing tumor conspicuity during primary staging. Meanwhile, color-coded iodine maps showed a significantly lower normalized iodine concentration in malignant tissue compared to a healthy myometrium. The study highlights the potential benefits of VMIs at 40 keV and iodine maps for improved visualization and assessment of endometrial adenocarcinoma during initial staging.

**Abstract:**

This study investigated whether virtual monoenergetic images (VMIs) and iodine mapping based on dual-energy CT (DECT) provide advantages in the assessment of endometrial cancer. A dual-source DECT was performed for primary staging of histologically proven endometrioid adenocarcinoma in 21 women (66.8 ± 12.0 years). In addition to iodine maps, VMIs at 40, 50, 60, 70, and 80 keV were reconstructed from polyenergetic images (PEIs). Objective analysis comprised the measurement of tumor contrast, contrast-to-noise ratio, and normalized iodine concentration (NIC). In addition, three radiologists independently rated tumor conspicuity. The highest tumor contrast (106.6 ± 45.0 HU) and contrast-to-noise ratio (4.4 ± 2.0) was established for VMIs at 40 keV. Tumor contrast in all VMIs ≤ 60 keV was higher than in PEIs (*p* < 0.001). The NIC of malignant tissue measured in iodine maps was substantially lower compared with a healthy myometrium (0.3 ± 0.1 versus 0.6 ± 0.1 mg/mL; *p* < 0.001). Tumor conspicuity was highest in 40 keV datasets, whereas no difference was found among PEIs and VMIs at 60 and 70 keV (*p* ≥ 0.334). Interobserver agreement was good, indicated by an intraclass correlation coefficient of 0.824 (0.772–0.876; *p* < 0.001). In conclusion, computation of VMIs at 40 keV and color-coded iodine maps aids the assessment of endometroid adenocarcinoma in primary staging.

## 1. Introduction

Endometrial cancer represents the most commonly diagnosed gynecological malignancy arising from the female reproductive tract in developed industrial nations, and the incidence rate of the condition is continuously increasing. The expected rate of newly diagnosed endometrial cancer was about 11,000 for Germany in 2022. Incidence increases with age, with an cumulated lifetime risk of about 1.9% in Germany and 1.7% in the USA [1]. Some risk factors, among others, include diabetes mellitus, metabolic syndrome, polycystic ovary syndrome, antihormonal treatment with tamoxifen and some hereditary syndromes like Lynch- and Cowden-syndrome. The risk also increases if the female sex hormone estrogen has a long-term effect on the body in increased quantities.

For evaluation of the local tumor extent, a transvaginal ultrasound is the first-line imaging technique. Whenever this method reveals ambiguous results, however, additional MRI is recommended for assessment of (deep) myometrial or cervical invasion. The latter results in upstaging to FIGO II, and, in cases of parametrial invasion, also in a change in the operative management. Another advantage of cross-sectional imaging lies in the simultaneous assessment of pelvic and paraaortic lymph node metastases, which also cause upstaging [2]. Screening for distant metastases usually comprises CT imaging of the chest and abdomen, including the pelvis. For locally advanced cancers, imaging is necessary to confirm the extent not only for the planning of cytoreductive surgery, but also for targeted radiation therapy. However, due to various potential contraindications, e.g., implanted cardiac pacemakers, claustrophobia or obesity, MRI may not be feasible in all patients [3]. Although preoperative imaging does not significantly change the management of patients with grades 1 and 2 endometrioid adenocarcinoma [4], this raises the general question of whether CT imaging can serve as an adequate replacement for assessing the local tumor spread in such situations [5].

With the emergence of dual-energy CT (DECT) scanners, which use X-ray spectra at both high and low energy levels to obtain more in-depth information on tissue properties, CT studies have become an alternative for multiple imaging tasks previously reserved for MRI. Established technical approaches for acquiring ‘spectral’ CT data include the use of fast kilovoltage switching, twin-beam acquisition, dual-layer detectors, and dual-source setups. Of note, the latter incorporates two X-ray tubes positioned approximately 90 degrees apart, which operate at different tube voltages. Each of the mostly vendor-specific techniques bears distinct advantages and limitations [6,7]. The common denominator of all DECT approaches, however, is the acquisition of polyenergetic images (PEIs), which can be used for a multitude of post-processing operations. One particularly interesting option lies in the spectral decomposition of different materials facilitated by the unique absorption behavior of each material at different energy levels. For oncologic imaging, the quantification of iodine concentration within a given target volume based on dedicated maps can reveal additional information regarding a tumorous lesion’s character, subsequently influencing treatment decisions [6,8]. Another clinically established benefit of DECT is the option to reconstruct virtual monoenergetic images (VMIs) at a certain keV level. As shown for various other cancer entities, low-keV VMIs close to the k edge of iodine provide markedly increased attenuation of enhancing structures and may improve tumor delineation in the process [9,10,11]. Despite these advantages, investigations focusing on the use of DECT in gynecological malignancies are scarce. While Rizzo et al. have previously used an earlier fast kV switching scanner to assess myometrial invasion of endometrial carcinoma [12], thorough investigations employing state-of-the-art radiation dose levels, multi-observer analyses and MRI correlation are lacking to the authors best knowledge.

Therefore, the purpose of the present study was to investigate in both a qualitative and quantitative fashion whether VMIs and iodine mapping based on datasets derived from a modern dual-source DECT are beneficial for primary staging of endometrioid adenocarcinoma.

## 2. Material and Methods

### 2.1. Patients

**Figure 1:**
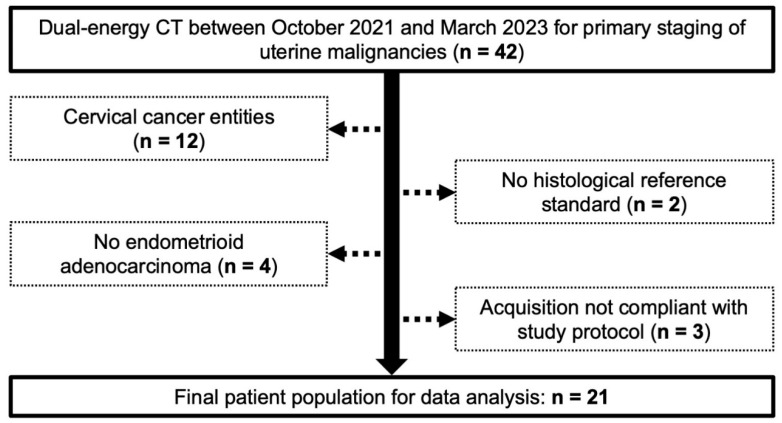
Flow chart for visualization of the study sample selection.

The local institutional review board approved this retrospective monocentric study and waived the need for additional written informed consent. Between October 2021 and March 2023, contrast-enhanced DECT for primary staging purposes in uterine malignancies was performed in 42 women at a tertiary-care university hospital. Study exclusion criteria comprised a definite diagnosis of cervical cancer, a lack of histopathological reference standards, the presence of endometrial cancer entities other than endometrioid adenocarcinoma, and/or deviation from the study-specific acquisition protocol. A flow chart depicting the study’s inclusion and exclusion criteria is provided in Figure 1.

### 2.2. Imaging

All scans were performed in a head-first supine position using a third-generation dual-source DECT with energy-integrating detector technology (SOMATOM Force, Siemens Healthineers, Erlangen, Germany). For each examination, 1 mL/kg bodyweight of an iodine-based contrast medium (Imeron 350, Bracco, Milan, Italy) was administered with a flow rate of at least 3 mL/s via an antecubital 18 G venous access. Once the signal intensity measurement in the abdominal aorta reached the predetermined threshold of 100 HU, arterial and portal venous phase scans started automatically, with a delay of 15 s and 50 s, respectively. In contrast to arterial phase imaging, portal venous scans were acquired with the scanner’s dual-energy mode using the following additional parameters: Tube voltage and reference current-time product of 90 kVp/160 ref. mAs (tube A) and Sn 150 kVp/100 ref. mAs (tube B), detector collimation of 128 × 0.6 mm; a rotation time of 0.5 s and helical pitch of 0.6. Automatic tube current modulation (CAREDose 4D, Siemens Healthineers) was activated by default. Scan volumes in the portal venous phase extended cranio-caudally from the liver dome to below the pubic bone.

Reconstruction of portal venous phase data was performed with a dedicated quantitative convolution kernel (Qr32, Siemens Healthineers), employing a slice thickness of 1.5 mm and a 512^2^ pixel matrix. Apart from being limited to a maximum diameter of 35.6 cm in spectral acquisition mode, the field of view was selected individually according to patient size. VMIs were reconstructed from PEI datasets with the same in-plane resolution and slice thickness at energy levels of 40, 50, 60, 70 and 80 keV. In addition, iodine maps of the pelvis were prepared for measurements of iodine concentration. For all post-processing applications, vendor-specific software was used (syngo.via VB40B, Siemens Healthcare GmbH, Erlangen, Germany). Additionally, while arterial phase images were also part of the clinical readings, these datasets were not included in the analysis of the present study.

### 2.3. Study Analysis

**Figure 2:**
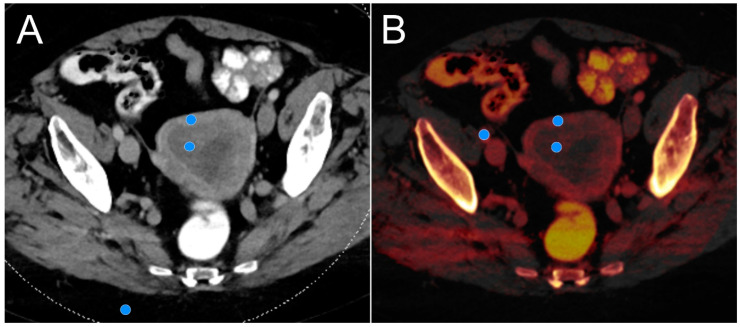
For calculation of tumor contrast and contrast-to-noise ratios, region-of-interest-based signal attenuation and noise measurements were performed in healthy myometrial tissue, endometrial cancer and subcutaneous gluteal fat for polyenergetic imaging (**A**) and each virtual monoenergetic dataset. Normalized iodine concentration was established based on regions of interest placed in the myometrium, tumor tissue and the external iliac artery on color-coded iodine maps (**B**).

For quantitative assessment, one reader with 10 years of training in gynecological oncology imaging positioned circular regions of interest (ROI) in the primary tumor, the tumor-free myometrium, and the gluteal subcutaneous fat tissue based on axial PEIs (Figure 2). Carefully avoiding vessel structures, fibrous tissue, and partial volume effects, the ROI were then copied to all five reconstructed VMIs to measure the mean signal attenuation and standard deviation thereof. In a second step, the ROI were copied to the respective iodine maps to record the actual iodine concentration within each tissue. Hereby, an additional ROI was drawn to assess the iodine concentration in the external iliac artery (EIA) for normalization purposes. The ROI size was preset to 10 mm^2^ and only adjusted when unavoidable (e.g., in vessels). In cases of small adenocarcinomas or low tumor contrast in PEI, a ROI placement was additionally correlated with MRI to avoid measurement in unrepresentative tissue. ROI-based measurements in PEIs and VMIs were used to calculate the tumor contrast ($\mathrm{Tumor}\;\mathrm{contrast}={\mathrm{mean}\;\mathrm{density}}_{\mathrm{myometrium}}-{\mathrm{mean}\;\mathrm{density}}_{\mathrm{tumor}}$) and contrast-to-noise ratio ($\mathrm{CNR}=\frac{\left({\mathrm{mean}\;\mathrm{density}}_{\mathrm{myometrium}}-{\mathrm{mean}\;\mathrm{density}}_{\mathrm{tumor}}\right)}{{\mathrm{noise}}_{\mathrm{fat}}}$). To account for variations in circulation time and scan duration among patients, the normalized iodine concentration (NIC) was calculated for tumorous (${\mathrm{NIC}}_{\mathrm{tumor}}=\frac{{\mathrm{iodine}\;\mathrm{concentration}}_{\mathrm{tumor}}}{{\mathrm{iodine}\;\mathrm{concentration}}_{\mathrm{EIA}}}$) and normal myometrial tissue (${\mathrm{NIC}}_{\mathrm{myometrium}}=\frac{{\mathrm{iodine}\;\mathrm{concentration}}_{\mathrm{myometrium}}}{{\mathrm{iodine}\;\mathrm{concentration}}_{\mathrm{EIA}}}$) based on the respective measurements in iodine maps [10].

To provide ancillary subjective evaluation, three board-certified radiologists with 10, 8, and 6 years of experience in the field independently analyzed the tumor conspicuity of each adenocarcinoma within each dataset on an equidistant 5-point scale (1 = low, 2 = fair, 3 = moderate, 4 = high, 5 = very high conspicuity). For their reads, observers were blinded to any clinical or scan protocol-related information. Datasets were presented separately and in randomized order. For determination of inter-reader reliability, the intraclass correlation coefficient (ICC) was computed based on absolute agreement of single measures in a two-way random effects scenario. ICC results were interpreted following established guidelines [13], with >0.90 = excellent, 0.75–0.90 = good, 0.50–0.75 = moderate, and <0.50 = poor inter-reader agreement.

### 2.4. Statistics

Statistical analyses were performed with dedicated software (SPSS Statistics 28, IBM, Armonk, NY, USA). Normal distribution of cardinal items was determined based on Shapiro–Wilk tests. Normally distributed data are reported as mean ± standard deviations, whereas median values with interquartile ranges are provided for non-parametric items. Depending on the scale level of each variable, either Friedman’s rank-based analysis of variance or a conventional repeated-measures ANOVA were employed to compare PEIs and the various VMI datasets. Additionally, *p* values of pairwise post hoc tests were corrected for multiple comparisons using the Bonferroni method. An alpha error below 0.05 was deemed indicative of statistical significance.

## 3. Results

### 3.1. Study Sample

Adhering to the predefined study inclusion and exclusion criteria, a total of 21 women with histopathologically proven endometroid adenocarcinoma were included in the data analysis. The mean age and BMI among patients were 66.8 ± 12.0 years (range 48–90 years) and 31.4 ± 6.0 kg/m^2^ (range 21.6–43.0 kg/m^2^), respectively. Tumors were classified as pathologic Grade 1 in 9.5% (n = 2), Grade 2 in 42.9% (n = 9), and Grade 3 in 47.6% (n = 10). The median time interval between DECT imaging and surgery was 20 days (interquartile range 10.5–34.5 days). Relevant patient and tumor data is comprised in Table 1.

### 3.2. Objective Lesion Conspicuity Assessment

**Figure 3:**
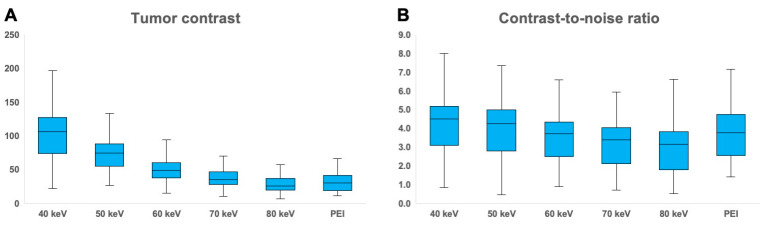
Boxplots illustrate differences in tumor contrast (**A**) and contrast-to-noise ratio (**B**) among virtual monoenergetic images from 40–80 keV and polyenergetic images (PEI).

**Figure 4:**
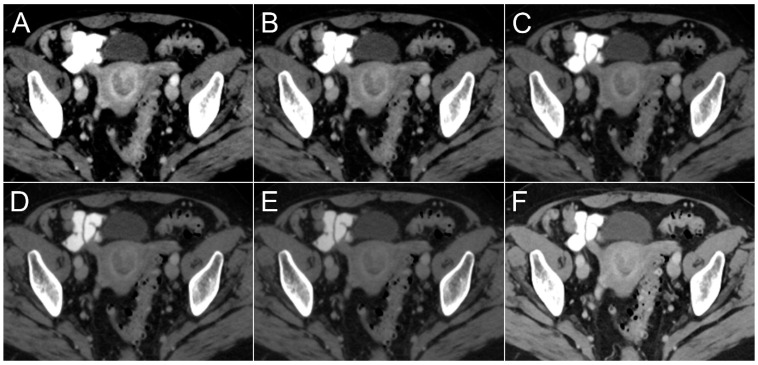
Exemplary axial slices on the level of the uterus visualize tumor contrast in virtual monoenergetic images at 40 keV (**A**), 50 keV, (**B**), 60 keV (**C**), 70 keV (**D**), 80 keV (**E**) and the corresponding polyenergetic images (**F**). In hypoattenuating endometrial cancer without myometrial invasion, hypervascularization of the intact subendometrial layer is best depicted in virtual monoenergetic reconstructions at 40 keV.

Figure 3 includes separate boxplot diagrams for visualization of differences in tumor contrasts and CNRs among VMIs and PEIs. The highest tumor contrast among datasets was established for virtual monoenergetic 40 keV reconstructions (106.6 ± 45.0 HU), whereas the lowest contrast was recorded for synthetic 80 keV images (28.3 ± 13.0 HU). Figure 4 comprises side-by-side visualizations of tumor contrasts with each VMI and the corresponding polyenergetic dataset in an endometrioid adenocarcinoma without myometrial invasion. Comparing monoenergetic and polyenergetic datasets in pairwise fashion, the endometrial tumor contrast determined in all VMIs ≤ 60 keV was higher than of PEIs (*p* < 0.001), while no significant difference was ascertained between PEIs and VMIs at 70 keV (*p* = 0.052) or 80 keV (*p* = 0.281). Accordingly, the CNR was highest in VMIs at 40 keV (4.4 ± 2.0) and lowest in VMIs at 80 keV (3.0 ± 1.4). However, unlike for tumor contrast, CNR comparisons revealed a significant advantage for PEIs over all monoenergetic reconstructions ≥70 keV (*p* ≤ 0.033) and no significant differences between PEIs and VMIs between 40–60 keV (*p* ≥ 0.999). Based on measurements in iodine maps, the NIC of endometrial cancer tissue (0.3 ± 0.1 mg/mL) was substantially lower compared with healthy myometrial tissue (0.6 ± 0.1 mg/mL; *p* < 0.001). The quantitative criteria of lesion conspicuity are summarized in Table 2.

### 3.3. Subjective Lesion Conspicuity Assessment

**Figure 5:**
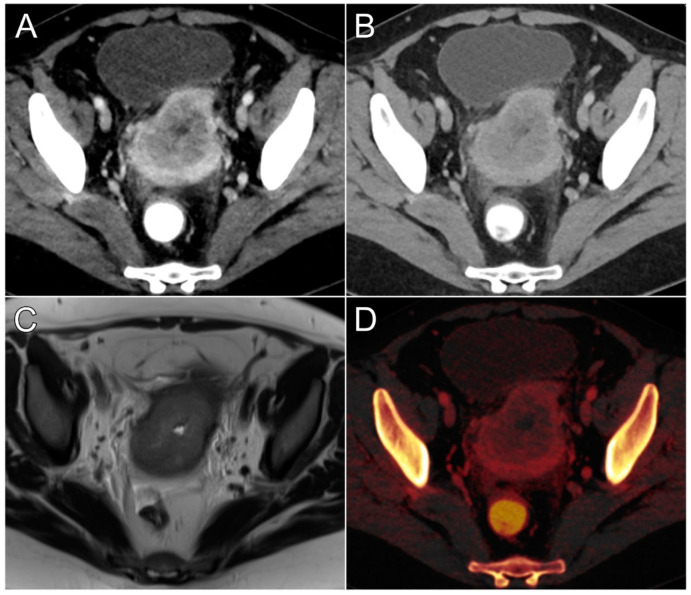
Primary staging of endometroid adenocarcinoma in a 53-year-old woman with improved assessability of myometrial invasion in a virtual monoenergetic reconstruction at 40 keV (**A**) compared to polyenergetic imaging (**B**). Tumor delineation in 40 keV images is even comparable to T2-weighted MRI (**C**) in this case. The additionally reconstructed iodine map (**D**) allows for quantitative assessment of iodine concentrations in tumors and healthy myometrial tissue.

Tumor conspicuity was deemed highest by the three observers in the virtual monoenergetic 40 keV (median value: 5 [interquartile range: 4–5]) and 50 keV datasets (4 [4–4.5]; *p* = 0.427). Meanwhile, the lowest conspicuity of endometrial carcinomas was determined for VMIs at 80 keV (2 [2–3]) and 70 keV (3 [2–3]; *p* = 0.064). No difference was found between PEIs and VMIs at either 60 keV (*p* = 0.334) or 70 keV (*p* > 0.999), whereas both 40 keV and 50 keV were considered to provide better tumor conspicuity than polyenergetic image stacks (both *p* < 0.001). A visual comparison of tumor delineation in VMIs with 40 keV and PEIs was combined with the corresponding iodine map and MRI for Figure 5. Interobserver agreement was good, as indicated by a single-measures ICC of 0.824 (95% confidence interval 0.772–0.876; *p* < 0.001). Table 3 contains a detailed display of subjective tumor conspicuity ratings.

## 4. Discussion

This study investigated the impact of virtual monoenergetic imaging and iodine maps on tumor delineation in primary dual-energy CT staging examinations for histopathologically proven endometrioid adenocarcinoma. Compared with standard polyenergetic datasets, the use of virtual monoenergetic images at 40 keV allowed for improved subjective tumor conspicuity and tumor-to-tissue contrast, as well as optimized contrast-to-noise ratios. As a major finding, a significant decrease in normalized iodine concentration was observed in hypoattenuating endometrial cancer tissue compared to the surrounding healthy myometrium.

In line with literature on other cancer entities [11,14,15], our results indicate that delineation of endometrial carcinomas improves with low-keV VMIs close to the k-edge of iodine (33.2 keV) due to more pronounced attenuation differences between hypovascularized tumor and hypervascularized myometrial tissue. One well-known drawback of low-keV reconstructions lies in their generally higher noise level resulting from the increasing influence of scattered low-energy X-ray photons [16,17]. While similar tendencies were found in our DECT-based stagings of endometrial cancer, we observed a 3.4-fold higher tumor contrast for VMIs at 40 keV compared with PEIs, resulting in almost identical CNRs in both datasets (4.4 vs. 4.2). Results from recent investigations on pancreatic cancer and hepatic tumors demonstrated similar results, with improved tumor contrasts and CNRs in low-keV reconstructions, suggesting high clinical impacts due to improved tumor detection rates [10,14,18]. At our institution, gynecological cancer patients are routinely scanned in dual-energy mode to allow for the reconstruction of virtual non-enhanced images instead of acquiring a true non-contrast phase of the pelvis. Therefore, the additional reconstruction of VMIs and iodine maps does not require much additional time and effort.

Whereas diagnostic sensitivity was not in the scope of the present study, all three radiologists’ perception of tumor conspicuity improved markedly when assessing VMIs at 40 and 50 keV. This finding is concordant with Rizzo et al., who postulated superior delineation of myometrial tumor invasion in 50 keV datasets. It must be stated, however, that 40 keV reconstructions were not evaluated in that study [12]. Adversely, the evaluation of monoenergetic images with higher keV levels entailed an inferior ability to assess the primary tumor, as no significant difference was ascertained for tumor contrasts between PEIs and VMIs ≥ 60 keV. Accordingly, subjective rating results yielded comparable but moderate tumor conspicuity among polyenergetic datasets (3 [3–3]) and VMIs with 60 keV (3 [3–4]) or 70 keV (3 [2–3]). Of note, we used a clinically established scan protocol with a portal venous phase acquired about 60 s after the intravenous administration of an iodine contrast agent, which represents a typical delay when aiming to evaluate the visceral organs [19]. In contrast, Rizzo et al. extended the delay before image acquisition to 120 s in their study, which conforms more to a nephrographic phase for full contrast of the renal glomeruli and collecting ducts. The images in the present study are therefore more likely to display the maximum contrast of the subendometrial layer, which is relevant for the depiction of a myometrial invasion [2].

Another technical advancement associated with spectral imaging via DECT is the option to perform material characterization and quantitative analyses of, for example, iodine uptake based on color-coded maps. While prior studies have demonstrated the clinical impact of this feature for the determination of malignancy in lymph nodes, such as in metastatic cervical squamous cell cancer [20], we evaluated the iodine concentration directly within the endometrial tumor tissue and compared it with a healthy myometrium. Additional normalization via reference measurements in the EIA has been described as outperforming simple iodine concentration measurements, thus resulting in more reliable and reproducible results [21,22]. Concordant with the results of Liang et al. for pancreatic adenocarcinomas, which constitute a similarly challenging imaging oncologic task, we reported a significantly lower NIC for the hypoattenuating tissue of endometrioid adenocarcinoma compared with the highly vascularized non-infiltrated myometrial tissue (−50%) [10]. Due to the limited size of the investigated patient sample, we advocate for future studies to investigate specifically whether reliable thresholds can be defined to assess the tumor extent and character solely based on iodine concentration in endometrium and myometrium.

As obesity is a well-established risk factor for the development of endometrial cancer, the evaluation of DECT as an alternative for primary staging of local tumor extent is important, particularly when considering the limited bore size and long examination times in MRI. Huflage et al. previously showed that abdominal dual-source DECT for staging purposes is feasible in obese patients when adhering to the limited dual-energy field of view [23]. While this can pose a challenge for the liver due to sheer organ size, volume and position of the uterus render insufficient dual-energy coverage highly unlikely. Notably, the patient size-dependent increase in radiation dose has been described to be less pronounced in DECT than in a single-energy CT [23]. Further benefits of using CT imaging include ubiquitous availability, rapid scan times, and the technique’s “one-stop-shop” potential by providing simultaneous local and distant staging. A more restrictive use of pelvic MRI in primary staging may allow for improved resource management, and MRI use could be reserved for specific imaging tasks such as ruling out myometrial cancer infiltration in women of childbearing age not wanting to undergo radical surgical treatment. With the recent emergence of photon-counting detector CT, which provides high spatial resolution and spectral post-processing options combined with superior dose efficiency in every scan [24,25], the importance of CT for staging of gynecological malignancies may increase even further in upcoming years.

Several limitations must be recognized when interpreting this study’s results. First, the single-center design with strict exclusion criteria (only primary stagings of endometroid adenocarcinomas were included) resulted in a relatively small patient sample. Considering the variable signal characteristics of different uterine malignancies, we prioritized homogeneity within our population over a less standardized cohort. With that being said, prospective evaluation of tumor detection rates and indicators of diagnostic accuracy in a larger sample would be desirable to confirm the clinical relevance of our results. In that regard, diagnostic sensitivity in particular should be assessed in systematic fashion. Second, since no inter-scanner or inter-vendor comparisons could be performed due to the retrospective patient enrollment, further investigations with multiple systems are necessary to determine if our results for dual-source DECT are reproducible with other technical approaches. Third, since an MRI was not available as a reference standard in all patients, the depth of myometrial invasion was not quantitatively assessed. While myometrial infiltration can be used as a surrogate parameter for lymph node involvement in the routine work-up of endometrial cancer, molecular analysis is increasingly used in clinical routine instead (e.g., by determination of mismatch repair, microsatellite instability, TP 53 and/or POLE status), having a major impact on treatment decision making in general and the preferred surgical approach in particular [26].

## 5. Conclusions

Spectral post-processing of dual-energy CT data improves subjective and objective tumor delineation in primary staging of endometrioid adenocarcinoma. While virtual monoenergetic images at 40 keV allow for improved conspicuity as well as superior tumor contrast and contrast-to-noise ratio, a significantly lower normalized iodine concentration could be ascertained in hypoattenuating endometrial cancer tissue based on color-coded perfusion maps.

## Figures and Tables

**Table 1:** Study population.

Sample Size	21 Women
Age [years]	66.8 ± 12
Size [cm]	164.9 ± 6.0
Weight [kg]	85.5 ± 17.0
BMI [kg/cm^2^]	31.4 ± 6.0
T stage T1a T1b T2 T3a T3b T4	7 (33.3%) 5 (23.8%) 2 (9.5%) 3 (14.3%) 3 (14.3%) 1 (4.8%)
Grading Grade 1 Grade 2 Grade 3	2 (9.5%) 9 (42.9%) 10 (47.6%)

**Table 2:** Quantitative image analysis.

	40 keV	50 keV	60 keV	70 keV	80 keV	PEI
Image noise [HU]	25.0 ± 5.2	22.8 ± 20.0	13.9 ± 2.8	11.4 ± 2.2	9.9 ± 1.9	7.8 ± 1.5
Tumor contrast [HU]	106.6 ± 45.0	75.5 ± 28.3	49.9 ± 21.1	36.8 ± 16.0	28.3 ± 13.0	31.7 ± 13.1
CNR	4.4 ± 2.0	4.1 ± 1.9	3.7 ± 1.4	3.3 ± 1.5	3.0 ± 1.4	4.2 ± 2.1
NIC_tumor_ [mg/mL]	0.3 ± 0.1					
NIC_myometrium_ [mg/mL]	0.6 ± 0.1					

Note—Results are displayed as mean values ± standard deviation. CNR—contrast-to-noise ratio; NIC—normalized iodine concentration; PEI—polyenergetic images.

**Table 3:** Subjective image assessment. Qualitative ratings for lesion conspicuity in virtual monoenergetic images (40–80 keV) and the polyenergetic image (PEI). Results are displayed as absolute numbers summarized from all three readers, with percentages in brackets. Median values are shown with interquartile ranges in parentheses.

Tumor Conspicuity	40 keV	50 keV	60 keV	70 keV	80 keV	PEI
Very high (5)	43 (68.3%)	16 (25.4%)	2 (3.2%)	0 (0%)	0 (0%)	0 (0%)
High (4)	18 (28.6%)	39 (61.9%)	24 (38.1%)	11 (17.5%)	4 (6.3%)	12 (19.0%)
Moderate (3)	2 (3.2%)	8 (12.7%)	35 (55.5%)	33 (52.4%)	19 (30.2%)	42 (66.7%)
Fair (2)	0 (0%)	0 (0%)	2 (3.2%)	19 (30.2%)	31 (49.2%)	9 (14.3%)
Low (1)	0 (0%)	0 (0%)	0 (0%)	0 (0%)	9 (14.3%)	0 (0%)
Median (IQR)	5 (4–5)	4 (4–4.5)	3 (3–4)	3 (2–3)	2 (2–3)	3 (3–3)

Note—Pooled rating results are displayed as absolute numbers with percentages in parentheses. Median values are given with interquartile ranges in parentheses. PEI—Polyenergetic images; IQR—Interquartile range.

## Data Availability

The datasets generated and/or analyzed during this study are not publicly available as DICOM data may contain patient information. Anonymized data can be obtained on reasonable request from the corresponding author.

## Abbreviations

CNR	contrast-to-noise ratio
DECT	dual-energy computed tomography
EIA	external iliac artery
ICC	intraclass correlation coefficient
NIC	normalized iodine concentration
PEI	polyenergetic imaging/images
ROI	region of interest
VMI	virtual monoenergetic imaging/images

## References

[1] Siegel R.L., Miller K.D., Fuchs H.E., Jemal A. (2022). Cancer statistics, 2022. CA Cancer J. Clin..

[2] Nougaret S., Horta M., Sala E., Lakhman Y., Thomassin-Naggara I., Kido A., Masselli G., Bharwani N., Sadowski E., Ertmer A. (2019). Endometrial Cancer MRI staging: Updated Guidelines of the European Society of Urogenital Radiology. Eur. Radiol..

[3] Torres M.L., Weaver A.L., Kumar S., Uccella S., Famuyide A.O., Cliby W.A., Dowdy S.C., Gostout B.S., Mariani A. (2012). Risk factors for developing endometrial cancer after benign endometrial sampling. Obstet. Gynecol..

[4] Lin M.Y., Dobrotwir A., McNally O., Abu-Rustum N.R., Narayan K. (2018). Role of imaging in the routine management of endometrial cancer. Int. J. Gynaecol. Obstet..

[5] Lalwani N., Dubinsky T., Javitt M.C., Gaffney D.K., Glanc P., Elshaikh M.A., Kim Y.B., Lee L.J., Pannu H.K., Royal H.D. (2014). ACR Appropriateness Criteria^®^ pretreatment evaluation and follow-up of endometrial cancer. Ultrasound Q..

[6] McCollough C.H., Leng S., Yu L., Fletcher J.G. (2015). Dual- and Multi-Energy CT: Principles, Technical Approaches, and Clinical Applications. Radiology.

[7] Adam S.Z., Rabinowich A., Kessner R., Blachar A. (2021). Spectral CT of the abdomen: Where are we now?. Insights Imaging.

[8] Hounsfield G.N. (2014). Computerized transverse axial scanning (tomography): Part 1. Description of system. Br. J. Radiol..

[9] De Cecco C.N., Darnell A., Rengo M., Muscogiuri G., Bellini D., Ayuso C., Laghi A. (2012). Dual-energy CT: Oncologic applications. AJR Am. J. Roentgenol..

[10] Liang H., Zhou Y., Zheng Q., Yan G., Liao H., Du S., Zhang X., Lv F., Zhang Z., Li Y.M. (2022). Dual-energy CT with virtual monoenergetic images and iodine maps improves tumor conspicuity in patients with pancreatic ductal adenocarcinoma. Insights Imaging.

[11] Nagayama Y., Tanoue S., Inoue T., Oda S., Nakaura T., Utsunomiya D., Yamashita Y. (2020). Dual-layer spectral CT improves image quality of multiphasic pancreas CT in patients with pancreatic ductal adenocarcinoma. Eur. Radiol..

[12] Rizzo S., Femia M., Radice D., Del Grande M., Franchi D., Origgi D., Buscarino V., Mauro A., Bellomi M. (2018). Evaluation of deep myometrial invasion in endometrial cancer patients: Is dual-energy CT an option?. Radiol. Med..

[13] Koo T.K., Li M.Y. (2016). A Guideline of Selecting and Reporting Intraclass Correlation Coefficients for Reliability Research. J. Chiropr. Med..

[14] Böning G., Feldhaus F., Adelt S., Kahn J., Fehrenbach U., Streitparth F. (2019). Clinical routine use of virtual monochromatic datasets based on spectral CT in patients with hypervascularized abdominal tumors—Evaluation of effectiveness and efficiency. Acta Radiol..

[15] Martin S.S., Wichmann J.L., Pfeifer S., Leithner D., Lenga L., Reynolds M.A., D’Angelo T., Hammerstingl R., Gruber-Rouh T., Vogl T.J. (2017). Impact of noise-optimized virtual monoenergetic dual-energy computed tomography on image quality in patients with renal cell carcinoma. Eur. J. Radiol..

[16] Bellini D., Gupta S., Ramirez-Giraldo J.C., Fu W., Stinnett S.S., Patel B., Mileto A., Marin D. (2017). Use of a Noise Optimized Monoenergetic Algorithm for Patient-Size Independent Selection of an Optimal Energy Level During Dual-Energy CT of the Pancreas. J. Comput. Assist. Tomogr..

[17] Hanson G.J., Michalak G.J., Childs R., McCollough B., Kurup A.N., Hough D.M., Frye J.M., Fidler J.L., Venkatesh S.K., Leng S. (2018). Low kV versus dual-energy virtual monoenergetic CT imaging for proven liver lesions: What are the advantages and trade-offs in conspicuity and image quality? A pilot study. Abdom. Radiol..

[18] Beer L., Toepker M., Ba-Ssalamah A., Schestak C., Dutschke A., Schindl M., Wressnegger A., Ringl H., Apfaltrer P. (2019). Objective and subjective comparison of virtual monoenergetic vs. polychromatic images in patients with pancreatic ductal adenocarcinoma. Eur. Radiol..

[19] Kulkarni N.M., Fung A., Kambadakone A.R., Yeh B.M. (2021). CT Techniques, Protocols, Advancements and Future Directions in Liver Diseases. Magn. Reson. Imaging Clin. N. Am..

[20] Tawfik A.M., Razek A.A., Kerl J.M., Nour-Eldin N.E., Bauer R., Vogl T.J. (2014). Comparison of dual-energy CT-derived iodine content and iodine overlay of normal, inflammatory and metastatic squamous cell carcinoma cervical lymph nodes. Eur. Radiol..

[21] Patel B.N., Vernuccio F., Meyer M., Godwin B., Rosenberg M., Rudnick N., Harring S., Nelson R., Ramirez-Giraldo J.C., Farjat A. (2019). Dual-Energy CT Material Density Iodine Quantification for Distinguishing Vascular From Nonvascular Renal Lesions: Normalization Reduces Intermanufacturer Threshold Variability. AJR Am. J. Roentgenol..

[22] Wu Y.Y., Wei C., Wang C.B., Li N.Y., Zhang P., Dong J.N. (2021). Preoperative Prediction of Cervical Nodal Metastasis in Papillary Thyroid Carcinoma: Value of Quantitative Dual-Energy CT Parameters and Qualitative Morphologic Features. AJR Am. J. Roentgenol..

[23] Huflage H., Kunz A.S., Hendel R., Kraft J., Weick S., Razinskas G., Sauer S.T., Pennig L., Bley T.A., Grunz J.P. (2023). Obesity-Related Pitfalls of Virtual versus True Non-Contrast Imaging-An Intraindividual Comparison in 253 Oncologic Patients. Diagnostics.

[24] Higashigaito K., Euler A., Eberhard M., Flohr T.G., Schmidt B., Alkadhi H. (2022). Contrast-Enhanced Abdominal CT with Clinical Photon-Counting Detector CT: Assessment of Image Quality Comparison with Energy-Integrating Detector, C.T. Acad. Radiol..

[25] Wrazidlo R., Walder L., Estler A., Gutjahr R., Schmidt B., Faby S., Fritz J., Nikolaou K., Horger M., Hagen F. (2023). Radiation Dose Reduction in Contrast-Enhanced Abdominal CT: Comparison of Photon-Counting Detector CT with 2nd Generation Dual-Source Dual-Energy CT in an oncologic cohort. Acad. Radiol..

[26] Corr B., Cosgrove C., Spinosa D., Guntupalli S. (2022). Endometrial cancer: Molecular classification and future treatments. BMJ Med..

